# Polarized and persistent Ca^2+^ plumes define loci for formation of wall ingrowth papillae in transfer cells

**DOI:** 10.1093/jxb/eru460

**Published:** 2014-12-10

**Authors:** Hui-Ming Zhang, Mohammad S. Imtiaz, Derek R. Laver, David W. McCurdy, Christina E. Offler, Dirk F. van Helden, John W. Patrick

**Affiliations:** ^1^School of Environmental and Life Sciences, University of Newcastle, Callaghan, NSW 2308,Australia; ^2^School of Biomedical Sciences and Pharmacy, University of Newcastle, Callaghan, NSW 2308,Australia

**Keywords:** Ca^2+^, signal, localized cell wall deposition, seed, *trans*-differentiation, transfer cell, wall ingrowth.

## Abstract

Transfer cell morphology is characterized by a polarized ingrowth wall comprising a uniform wall upon which wall ingrowth papillae develop at right angles into the cytoplasm. The hypothesis that positional information directing construction of wall ingrowth papillae is mediated by Ca^2+^ signals generated by spatiotemporal alterations in cytosolic Ca^2+^ ([Ca^2+^]_cyt_) of cells *trans*-differentiating to a transfer cell morphology was tested. This hypothesis was examined using *Vicia faba* cotyledons. On transferring cotyledons to culture, their adaxial epidermal cells synchronously *trans*-differentiate to epidermal transfer cells. A polarized and persistent Ca^2+^ signal, generated during epidermal cell *trans*-differentiation, was found to co-localize with the site of ingrowth wall formation. Dampening Ca^2+^ signal intensity, by withdrawing extracellular Ca^2+^ or blocking Ca^2+^ channel activity, inhibited formation of wall ingrowth papillae. Maintenance of Ca^2+^ signal polarity and persistence depended upon a rapid turnover (minutes) of cytosolic Ca^2+^ by co-operative functioning of plasma membrane Ca^2+^-permeable channels and Ca^2+^-ATPases. Viewed paradermally, and proximal to the cytosol–plasma membrane interface, the Ca^2+^ signal was organized into discrete patches that aligned spatially with clusters of Ca^2+^-permeable channels. Mathematical modelling demonstrated that these patches of cytosolic Ca^2+^ were consistent with inward-directed plumes of elevated [Ca^2+^]_cyt_. Plume formation depended upon an alternating distribution of Ca^2+^-permeable channels and Ca^2+^-ATPase clusters. On further inward diffusion, the Ca^2+^ plumes coalesced into a uniform Ca^2+^ signal. Blocking or dispersing the Ca^2+^ plumes inhibited deposition of wall ingrowth papillae, while uniform wall formation remained unaltered. A working model envisages that cytosolic Ca^2+^ plumes define the loci at which wall ingrowth papillae are deposited.

## Highlight

A persistent and polarized cytosolic Ca^2+^ signal, formed into plumes by co-operative activities of plasma membrane Ca^2+^ channels and Ca^2+^-ATPase clusters, directs papillate wall ingrowth deposition in *trans*-differentiating transfer cells.

## Introduction

Cytosolic Ca^2+^ is a conserved signal directing polarized cell development in algae (Wheeler and Brownlee, 2008), animals (Tojima, 2012), fungi (Brand and Gow, 2009), and plants (Kudla *et al.*, 2010). For plants, the most studied experimental cell models in which cytosolic Ca^2+^ functions as a polarity signal are elongating pollen tubes (Hepler *et al.*, 2012) and root hairs (Cárdenas, 2009). In these cells, a tip-high gradient of [Ca^2+^]_cyt_ directs polarized delivery of vesicles containing cargoes of cell wall building material for continued tip growth. The polarized vesicle delivery depends upon a Ca^2+^-induced remodelling of the actin cytoskeleton combined with alterations to the secretory apparatus (Cárdenas, 2009; Michard *et al.*, 2009). Distinct spatiotemporal patterns of cytosolic Ca^2+^ signals encrypt regulatory information (Kudla *et al.*, 2010; Hepler *et al.*, 2012). The distinctive patterns arise from the co-operative activities of Ca^2+^-permeable channels releasing Ca^2+^ into the cell cytosol from extracellular and intracellular sources and Ca^2+^ retrieval back into these compartments by Ca^2+^-ATPases and Ca^2+^/proton antiporters (McAinsh and Pittman, 2009; Hepler *et al.*, 2012).

In contrast to tip growth of pollen tubes and root hairs, there is a dearth of studies addressing signalling mechanisms regulating polarized deposition of wall thickenings in mature cells such as stomatal guard (Apostolakos *et al.*, 2009) and transfer (Andriunas *et al.*, 2013) cells. Transfer cells are a subset of plant cells that *trans*-differentiate from pre-existing cell types. Their wall thickenings (ingrowth walls), often polarized, are comprised of a complex labyrinth of invaginated wall ingrowths arising initially as discrete papillae from an underlying uniform wall (McCurdy *et al.*, 2008). Collectively, wall ingrowths provide a scaffold to support a greatly amplified surface area of transporter-enriched plasma membrane. This structure/function configuration confers on transfer cells the capacity to support high rates of apo/symplasmic solute exchange (Offler *et al.*, 2003) that translates into regulating resource allocation between competing organs and hence contributing to the realization of crop yield potential (Andriunas *et al.*, 2013). Therefore, discovering mechanisms regulating deposition of transfer cell ingrowth walls, and in particular their wall ingrowths, not only is of intrinsic biological interest but also offers opportunities to engineer increases in crop yield.

There are compelling technical challenges contributing to the paucity of information available on regulatory mechanisms controlling deposition of the ingrowth wall of transfer cells. Foremost amongst these is that transfer cells normally occur in low numbers embedded deep within tissues. This challenge is circumvented by adaxial epidermal cells of *Vicia faba* cotyledons that form ingrowth walls rapidly and synchronously within hours of the cotyledons being placed in culture (Wardini *et al.*, 2007). Several thousand adaxial epidermal cells are readily accessible for visualization and experimental manipulation, enabling transfer cell induction to be studied with relative ease (Zhou *et al.*, 2010). Significantly, these culture-induced adaxial epidermal transfer cells *trans*-differentiate to a transfer cell morphology and function comparable with their *in planta* abaxial counterparts (Farley *et al.*, 2000).

Studies using this *V. faba* cotyledon system have discovered components of an epidermal-cell-specific network of signalling molecules that regulate assembly of an ingrowth wall. Upon cotyledon transfer to culture, an epidermal-cell-specific spike in auxin levels (Dibley *et al.*, 2009) induces an ethylene signal, transduced through the Ethylene Insensitive 3 pathway (Zhou *et al.*, 2010), antagonistically modulated by a converging intracellular glucose signalling pathway (Andriunas *et al.*, 2011). The regulatory influence of ethylene on ingrowth wall assembly is mediated, in part, by ethylene-induced expression of two respiratory burst oxidases (Andriunas *et al.*, 2012; Xia *et al.*, 2012). These catalyse the generation of extracellular hydrogen peroxide (H_2_O_2_) that localizes to the outer periclinal walls of the epidermal cells (Andriunas *et al.*, 2012; Xia *et al.*, 2012). The extracellular H_2_O_2_ signal activates cell wall biosynthesis and provides a positional cue directing polarized deposition of the uniform wall (Andriunas *et al.*, 2012; Xia *et al.*, 2012). What is currently unclear is the identity of signal(s) directing construction of localized wall ingrowth papillae that represent the first phase in the development of the complex wall ingrowth labyrinth.

Using *V. faba* cotyledon culture, in combination with live cell imaging and computational modelling, it was discovered that polarized and persistent plumes of cytosolic Ca^2+^ are formed within the *trans-*differentiating epidermal cells. Co-operative activities of ordered clusters of plasma membrane Ca^2+^-permeable channels surrounded by Ca^2+^-ATPases are responsible for generating Ca^2+^ plumes. These are shown to provide loci at which wall ingrowth papillae are deposited.

## Materials and methods

### Plant growth and cotyledon culture conditions

Developing seeds were harvested from *V. faba* L. (cv. Fiord) plants raised under controlled environmental conditions. Cotyledons were surgically removed from their seed coats and prepared for aseptic culture on a Murashige and Skoog (MS) medium (Murashige and Skoog, 1962) as previously described (Zhou *et al.*, 2010).

### Visualizing Ca^2+^ signals and fluorescently labelled Ca^2+^-permeable channels by confocal laser scanning microscopy

Cotyledons were pre-loaded with the single wavelength Ca^2+^-sensitive fluorescent probe, Oregon Green 488 BAPTA-1-acetoxymethyl (AM) ester (Invitrogen, USA) following a protocol adapted from Zhang *et al.* (1998). During probe loading, cotyledons were incubated in 20 μM Oregon Green BAPTA 1-AM ester in MS medium for 3h at 4 °C to minimize AM ester hydrolysis by extracellular esterases. Cotyledons were then transferred to liquid MS medium for 2h at 26 °C to energize cleavage of loaded AM ester by cytosolic esterases, thereby trapping the impermeable Oregon Green dye in the cytosol of viable epidermal cells (see Supplementary Fig. S1 available at *JXB* online). To visualize the cellular distribution of Ca^2+^-permeable channels, cotyledons were stained with 600nM DM-BODIPY(–)-dihydropyridine (fl-DHP; Invitrogen, USA) in MS medium for 2h at 20 °C (Furch *et al.*, 2009). Viable epidermal cells were identified in hand-cut sections of Oregon Green-pre-loaded or fl-DHP-stained cotyledons by floating the sections for 20min on 0.1% (w/v) tetrazolium blue in phosphate-buffered saline (PBS) plus 100mM sucrose. In specified instances, sections were counterstained with 0.1% (w/v) Calcofluor White for 30 s to label the walls of adaxial epidermal cells or loaded with 8-acetoxypyrene-l,3,6-trisulphonic acid, trisodium salt (HPTS-acetate). Thereafter, sections were transferred to 1ml of 100mM sucrose/PBS in a bathing ring and visualized by confocal microscopy.

Multichannel imaging of cotyledon sections was performed using an Olympus FV1000 confocal laser scanning microscope (Olympus, Japan). Calcofluor White was excited with a 405nm UV laser (50 mW, laser power set to 15%) and emitted fluorescence collected at 440–490nm, while Oregon Green, fl-DHP, and HPTS were excited with a 473nm diode laser (15 mW, laser power set to 50%) and their emitted fluorescence captured at 510–550nm. Gain of the photomultiplier tube was set to 500V for Calcofluor White and to 700V for Oregon Green, fl-DHP, or HPTS. Cotyledon sections were observed with a ×60 oil-immersion lens. Real-time intensity changes in Oregon Green fluorescence were recorded using a Hamamatsu™ spinning disc system coupled to a Zeiss confocal microscope (Zeiss, Germany) with a ×20 air objective, a 488nm argon laser (20 mW laser power set to 40%), and 488/515nm emission filters.

To identify the subcellular localization of the Ca^2+^ signal and Ca^2+^-permeable channels, Oregon Green-loaded or fl-DHP-stained cotyledons were counterstained with 20 μM RH-414, a plasma membrane marker (Molecular Probes), during the last 30min of cleaving Oregon Green ester or fl-DHP staining. Thereafter, cotyledon hand sections were floated for 20min on MS medium containing 0.1% (w/v) tetrazolium blue (cell viability) with their osmolalities adjusted to 300 mOsmol Kg^–1^ (turgid cells) or 500 mOsmol Kg^–1^ (plasmolysed cells) using betaine. Cell walls were stained with 0.1% (w/v) Calcofluor White. A 559nm diode laser (15 mW, laser power set to 25%) with a 625–725nm emission filter was used to visualize RH-414 fluorescence (gain of the photomultiplier tube was set to 500V). Spectrum settings for Calcofluor White, Oregon Green, and fl-DHP were as indicated previously.

Relative estimates of [Ca^2+^]_cyt_ were obtained by constructing a calibration curve from pixel intensities of Oregon Green fluorescence in epidermal cells of cotyledons equilibrated in a 10–1000nM clamped range of extracellular Ca^2+^ concentrations using CALBUF-2 buffer (WPI, USA). Extra-/intracellular equilibration of Ca^2+^ was imposed 10min prior to confocal observation by permeabilizing and depolarizing membrane potentials of the epidermal cells by incubating the tissue sections in MS medium containing 10 μM A23187 and 10 μM CCCP, respectively. Thereafter, Oregon Green fluorescence of epidermal cells was captured by confocal microscopy as previously described.

### Electron microscopy

Ingrowth walls of epidermal cells were visualized in cotyledon sections prepared for transmission electron microscopy. Tissue wedges, surgically removed from cultured cotyledons, were fixed and embedded in London Resin White resin (Offler *et al.*, 1997). Ultrathin (60nm thick) transverse sections were stained with saturated uranyl acetate and counterstained with 1% (w/v) lead citrate, prior to viewing with a JEOL 1200 EX II transmission electron microscope (JEOL, Japan). Wall ingrowth papillae on cytosolic faces of outer periclinal walls of fractured epidermal peels were prepared for observation using a Phillips XL30 scanning electron microscope (Phillips, The Netherlands) as described in Zhou *et al.* (2010).

### Mathematical modelling

A mathematical model was formulated to produce a two-dimensional microdomain model. Ca^2+^ influx channels and efflux pumps were placed at various locations along the hypothetical plasma membrane and the model simulated until steady state was reached. Ca^2+^ flux rates and numbers of Ca^2+^ channels/pumps were balanced to ensure the model reached steady-state concentrations. The steady-state intracellular [Ca^2+^]_cyt_ distribution pattern was compared with that observed experimentally. This process was iterated until a best fit of the numerical and experimental pattern was reached.

Equations formulating the system are given below:

$$\frac{\partial c}{\partial t}=\frac{\alpha (x)F_{in}-\beta (x)F_{out}}{A_{vox}}+D_{c}\frac{\partial^{2}c}{\partial x^{2}}$$ (1)

$$F_{out}=F_{max}\frac{c}{c+k}$$ (2)

$$\alpha (x)=\{\begin{array}{ll} 1, & x\;contains\;influx\;channels\\ 0, & x\;contains\;no\;pumps \end{array}$$ (3)

$$\beta (x)=\{\begin{array}{ll} 1, & x\;contains\;efflux\;pumps\\ 0, & x\;contains\;no\;pumps \end{array}$$ (4)

where *C* is the concentration of the Ca^2+^ signal, *F*_*in*_=8e-3nM s^–1^ and *F*_*max*_=1e-1nM s^–1^, are Ca^2+^ influx and efflux rates, respectively, and *K*=1000nM is the Ca^2+^ concentration supporting the half-maximal rate of Ca^2+^ transport through the influx channel (i.e. *K*_*m*_). Initial conditions and parameter values for the influx channels and efflux pumps were chosen arbitrarily as only the final experimental steady-state concentration pattern is critical to this study. In Equation 1, a diffusion coefficient *D*_*c*_=1e-9 m^2^ s^–1^ accounts for Ca^2+^ diffusion within a plant cell cytosol (Thomas, 1982). Space was divided into an *A*_*vox*_=0.1 μm^2^ meshing area. No flux boundary conditions were defined, and the simulation was carried out in Matlab (Natick, USA) using variable step stiff ode solver ode15s.

### Data analyses

**Fig. 1.**
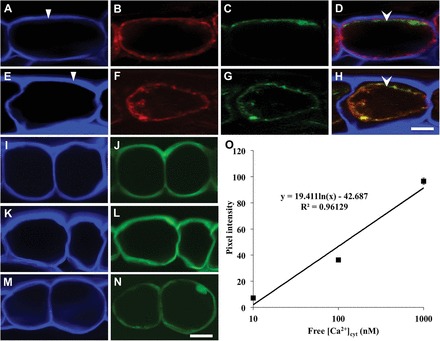
Validating Oregon Green 488 BAPTA-1 AM ester fluorescence as a relative measure of [Ca^2+^]_cyt_ in epidermal cells of cultured cotyledons. Confocal laser scanning microscope images of transverse sections of epidermal cells (A–N). Turgid (A–D) and plasmolysed (E–H) adaxial epidermal cells. Their cell walls are visualized (Calcofluor White; A, E; darts indicate the outer periclinal wall), plasma membranes (RH-414 fluorescence; B, F), and Ca^2+^ signal (Oregon Green fluorescence; C, G) together with their image overlays (D, H; arrowhead indicating Oregon Green fluorescence). Note that the protrusions into the cytoplasm of the plasmolysed cells labelled with Oregon Green and RH-414 (F, G, H) are likely to be infolded portions of the plasma membrane formed as the protoplast volume shrinks during plasmolysis. Distribution of Oregon Green (J, L) or HPTS (N) dye in Calcofluor White-co-stained tissue sections (I, K, M respectively) floated for 10min on medium alone (N) or medium containing 10 μM A23187 (J) or 500nM Eosin Yellow (L). Note that HPTS also loaded into nuclei of epidermal cells as found by Wright and Oparka (1996). Scale bar=10 μm for A–H and 20 μm for I–N. Calibration curve (O) of Oregon fluorescence (pixel intensity) with intracellular Ca^2+^ concentrations of epidermal cells permeabilized with A23187/CCCP and equilibrated in buffered bath concentrations of Ca^2+^.

For visualization of Oregon Green fluorescence, images captured by the Olympus FV1000 confocal microscope were converted and analysed in FV10-ASW 4.0 viewer. Time-course data of Oregon Green fluorescence intensity were analysed by Imaging Workbench 6.0 software. Pixel intensities of Oregon Green, fl-DHP, RH-414, and HPTS fluorescence were corrected for background by subtracting fluorescence intensities measured in the inner and outer periclinal regions of the epidermal cells that were not loaded/stained with the dyes. Relative estimates of [Ca^2+^]_cyt_ are reported as arbitrary units derived from a fitted calibration curve (Fig. 1O).

To detect bright spots of fl-DHP or Oregon Green fluorescence in paradermal confocal images, the raw images were filtered and intensity peaks detected. A computerized algorithm was run to fit a two-dimensional Gaussian around each detected peak, given that bright punctate fluorescent spots are well represented by a point spread function. Fits with adjusted *R* ^2^ >0.8 were accepted and the sigma value used as an indicator of diameter. The software drew a calculated diameter around each detected fluorescent patch, which was then visually inspected for errors (Supplementary Fig. S4 at *JXB* online). Thus the algorithm provided a methodical non-biased detection of circular bright spots against noise in the images.

The percentages of cells with wall ingrowth papillae were obtained by scoring the presence/absence of wall ingrowth papillae in scanning electron microscopy images of epidermal peels (Zhou *et al.*, 2010). The cell wall thicknesses of adaxial epidermal cells, visualized in transmission cross-sections, were estimated from determining cell wall surface areas expressed on a length basis (i.e. nm^2^ nm^–1^=nm) using ImageJ software. Cytoplasmic volumes of inner and outer periclinal regions of epidermal cells were estimated as the product of their cytoplasmic widths using the same protocol as for wall widths (see above) and cell surface areas determined from scanning electron micrographs of epidermal peels.

Statistical significance of treatment effects was determined using *t*-test in Microsoft Excel 2007.

## Results

### Confocal imaging of cytosolic Ca^2+^ in cotyledon epidermal cells

Compared with epifluorescence microscopy, optical sectioning by confocal microscopy of thick (100 μm) hand sections of cotyledons was found to capture clear fluorescence images of *trans*-differentiating epidermal cells (see Supplementary Fig. S1 at *JXB* online). In the absence of a stable or transient transformation system for *V. faba* to introduce Ca^2+^ reporters (Swanson *et al.*, 2011), confocal imaging fluorescence of a pre-loaded single wavelength Ca^2+^-sensitive fluorescent probe, Oregon Green 488 BAPTA-1 AM ester, was relied on to test whether [Ca^2+^]_cyt_ gradients develop within the *trans*-differentiating epidermal cells. A band of Oregon Green fluorescence was localized to the outer periclinal region of metabolically competent epidermal cells (Supplementary Fig. S1F versus H) loaded with AM ester of the dye under low temperature (Supplementary Fig. S1F versus B).

It was not possible to undertake pseudo ratiometric analysis of the Oregon Green fluorescence as AM esters of the reference dyes, Fura-Red or Texas Red (Swanson *et al.*, 2011), could not be loaded into epidermal cells. Thus deducing Ca^2+^ signal dynamics from Oregon Green fluorescence depended on there being no differences in intracellular dye concentrations and optical path lengths as well as no subcellular localization and cellular compartmentation of the dye (Swanson *et al.*, 2011). These issues were evaluated in a series of experiments as outlined below.

**Fig. 2.**
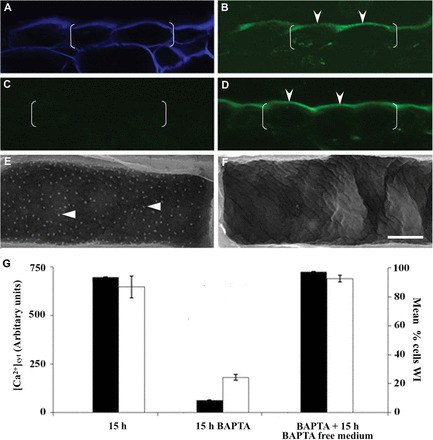
Relationship between Ca^2+^ signal and formation of wall ingrowth papillae. (A–D) Confocal laser scanning images of transverse sections of cotyledons cultured for 15h on MS medium alone (A, B), MS medium containing 600 μM BAPTA (C), or thereafter transferred to MS medium alone for a further 15h (D). Cell walls are visualized by staining with Calcofluor White (A) and the same section showing Oregon Green fluorescence (B). Epidermal cells are bracketed and Oregon Green fluorescence is labelled with arrowheads (B, D). (E, F) Scanning electron microscope images of cytoplasmic faces of outer periclinal walls of epidermal cells of cotyledons cultured for 15h in the absence (E) or presence (F) of 600 μM BAPTA. Examples of wall ingrowth papillae are labelled with darts. Scale bar = 20 µm for A to D and 5 µm for E and F. (G) Relative estimates of [Ca^2+^]_cyt_ (filled columns) and percentages of adaxial epidermal cells containing wall ingrowth papillae (WIs; empty columns) following cotyledon culture on media described in A–F. Data represent the mean± SEM. Relative estimates of [Ca^2+^]_cyt_ based on determining Oregon Green fluorescence in 100 cells from four cotyledons, 20–30 cells per cotyledon. The percentages of cells with wall ingrowth papillae were determined from observations of 100 cells per cotyledon across six replicates.

The cellular location of the Oregon Green fluorescent band was determined by co-staining hand sections with Calcofluor White (cell wall; Fig. 1A, E) and the plasma membrane tracker RH-414 (Fig. 1B, F). Image overlays showed that, in turgid and plasmolysed epidermal cells, Oregon Green fluorescence (Fig. 1C, G) was located on the cytoplasmic side of the plasma membrane (Fig. 1D, H, respectively). For plasmolysed epidermal cells, Oregon Green fluorescence, and hence the reporter dye, was dispersed around the entire cytoplasm (Fig. 1G, H). This Oregon Green distribution pattern was also detected in turgid epidermal cells permeabilized with the Ca^2+^ ionophore A23187 (Fig. 1I, J; Supplementary Table S1 at *JXB* online) and in cells treated with Eosin Yellow to block cytosolic Ca^2+^ efflux by inhibiting plasma membrane Ca^2+^-ATPases (Fig. 1K, L; Supplementary Table S1). A corresponding even distribution of a non-Ca^2+^-sensitive and membrane-impermeant fluorophore, HPTS (Wright and Oparka 1996; see Fig. 1M, N; Supplementary Table S1), confirmed an absence of any localized intracellular dye accumulation consistent with no detectable differences in subcellular cytoplasmic volumes within the epidermal cells (Supplementary Table S1). Organelle compartmentation of Oregon Green was considered unlikely as: (i) fluorescence was absent from anticlinal and inner periclinal cytoplasmic regions (Figs 1C, 2B); and (ii) the outer periclinal fluorescent band was reduced to background when Ca^2+^ influx into cells was blocked (Fig. 2B versus C). Effects of uneven tissue section geometries altering optical path lengths and hence fluorescent intensities were minimized by replicated measures of Oregon Green fluorescence intensities (Supplementary Table S1). Finally, Oregon Green fluorescence was not detectable in epidermal cells of freshly harvested cotyledons (Supplementary Fig. S1B versus F). This suggests that the cytosolic Ca^2+^ signal was induced developmentally rather than from wounding on cutting hand sections. Collectively these findings indicate that the epidermal-cell-specific and polarized Oregon Green fluorescent band (Fig. 1C; Supplementary S1F), induced during cotyledon culture (Supplementary Fig. S1F versus B), resulted from a polarized intracellular elevation in [Ca^2+^]_cyt_ detected by a uniform dye distribution throughout the cytosol of each epidermal cell.

Intensities of Oregon Green fluorescence, measured as pixel intensities, provided relative estimates of [Ca^2+^]_cyt_ as shown by equilibrating A23187-permeabilized epidermal cells across the known range of intracellular Ca^2+^ concentrations (Fig. 1O; Furch *et al.*, 2009; Swanson *et al.*, 2011). Thus, throughout the remainder of the text, relative [Ca^2+^]_cyt_ values are derived from pixel intensity measures of Oregon Green fluorescence.

### An epidermal cell-specific and polarized cytosolic Ca^2+^ signal is essential for formation of wall ingrowth papillae

The cytosolic Ca^2+^ signal in epidermal cells (Figs 1D, 2B versus A) co-localized with the site of deposition of wall ingrowth papillae on the cytoplasmic face of their outer periclinal walls (Fig. 2E). A causal relationship between the Ca^2+^ signal and formation of wall ingrowth papillae is suggested by the 93% BAPTA suppression of [Ca^2+^]_cyt_ (Fig. 2C versus B, G) coinciding with a 75% reduction in cells forming wall ingrowth papillae (Fig. 2F versus E, G). The causality of this relationship was verified by finding that BAPTA suppression of [Ca^2+^]_cyt_ and formation of wall ingrowth papillae was reversed upon transferring cotyledons to a BAPTA-free medium containing 3mM Ca^2+^ (Fig. 2D versus C, G). Together, these observations are consistent with a cytosolic Ca^2+^ signal, originating from an extracellular source, directing deposition of wall ingrowth papillae.

### Generation of the polarized and persistent cytosolic Ca^2+^ signal depends upon the co-operative activity of Ca^2+^-permeable channels and Ca^2+^-ATPases

The dependence of the cytosolic Ca^2+^ signal intensity upon an extracellular Ca^2+^ source (Fig. 2B versus C, G) suggests that it was generated by an inward-directed Ca^2+^ flux through plasma membrane Ca^2+^-permeable channels. This proposition was supported by a significant dampening of [Ca^2+^]_cyt_ when cotyledons were cultured in gadolinium, a blocker of plasma membrane-located Ca^2+^-permeable channels (Table 1). In contrast, blocking Ca^2+^-sensitive IP3, ryanodine, or cyclic ADP-ribose receptor Ca^2+^-permeable channels located on endomembranes with 2-APB, ryanodine, or ruthenium red, respectively (Peiter, 2011), exerted no effect on [Ca^2+^]_cyt_ (Table 1). Collectively, these findings suggest that influx of Ca^2+^ through Ca^2+^-permeable channels, located on the plasma membrane, accounted for the observed elevation of [Ca^2+^]_cyt_ in the *trans*-differentiating epidermal cells. Exposure of cultured cotyledons to nifedipine and verapamil attenuated [Ca^2+^]_cyt_ (Table 1). These responses indicated that these plasma membrane Ca^2+^-permeable channels are L-type voltage-dependent and non-selective cation channels (Demidchik and Maathuis, 2007). It is not known at this stage whether these channels belong to the cyclic nucleotide-gated channel and glutamate receptor-like channel families active in contributing to tip-high Ca^2+^ signals in elongating pollen tubes (Hepler *et al.*, 2012). However, consistent with the cytosolic Ca^2+^ signal directing formation of wall ingrowth papillae, [Ca^2+^]_cyt_ and formation of wall ingrowth papillae exhibited similar proportionate responses to these Ca^2+^ channel blockers (Table 1).

**Table 1.** Effects of Ca^2+^ channel blockers on the formation of a Ca^2+^ signal and wall ingrowth (WI) papillaeRelative estimates of [Ca^2+^]_cyt_ are based on determining Oregon Green fluorescence in 100 cells from four cotyledons, 20–30 cells per cotyledon. The percentages of cells with wall ingrowth papillae were derived from observations of 100 cells per cotyledon across six replicates. Percentage inhibition (relative to the control) is presented in parentheses.

Cotyledon treatment	[Ca^2+^]_cyt_ (arbitary units)	% of cells with WIs
Control	680±22	88.5±1.0
Gadolinium (1mM)	19±1 (97)	16.7±4.0 (81)
2-APB (100 μM)	654±24 (4)	90.6±1.4 (0)
Ryanodine (100 μM)	678±25 (0)	90.0±0.6 (0)
Ruthenium red (1mM)	644±24 (5)	91.1±0.7 (0)
Verapamil (200 μM)	65±2 (90)	31.7±2.3 (64)
Nifedipine(100 μM)	67±3 (90)	34.5±2.0 (61)

Data represent the mean ±SEM.

Whether the polarized cytosolic Ca^2+^ signal resulted from an asymmetric distribution of plasma membrane Ca^2+^-permeable channels within the epidermal cells was evaluated cytochemically using a fluorescent nifedipine analogue, fl-DHP, that binds to nifedipine-sensitive Ca^2+^ channels (Vallée *et al.*, 1997). The activities of these channels accounted for 90% of the elevation in [Ca^2+^]_cyt_ (Table 1). To determine the intracellular localization of bound fl-DHP, hand sections of cotyledons were counterstained with Calcofluor White (cell wall) and the plasma membrane tracker RH-414 (Supplementary Fig. S2 at *JXB* online). Image overlays of turgid and plasmolysed epidermal cells indicated that fl-DHP fluorescence localized to the outer perimeter of their protoplasts (Supplementary Fig. S2D, H). That fl-DHP bound to Ca^2+^-permeable channels in this location was supported by competition with non-labelled nifedipine substantially reducing the fluorescence intensity of, and hence binding by, fl-DHP (Supplementary Fig. S3B versus D; Supplementary Table S2).

**Fig. 3.**
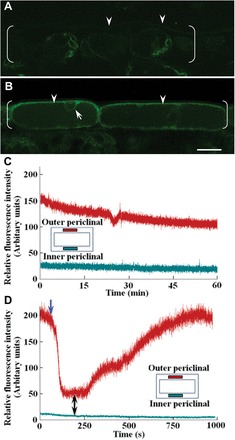
Intracellular distribution of Ca^2+^-permeable channels and temporal changes in [Ca^2+^]_cyt_. (A, B) Confocal laser scanning images of transverse sections of freshly harvested (A) and 15h cultured (B) cotyledons stained with fl-DHP to detect Ca^2+^-permeable channels. fl-DHP fluorescence localized to putative plasma membrane lining the outer periclinal region (arrowheads in A, B) and to endomembranes lining the inner cytoplasmic face of the nucleus (arrow in B) of each adaxial epidermal cell (bracketed). Scale bar=25 μm. (C, D) Real-time measures of relative [Ca^2+^]_cyt_, detected by spinning-disc confocal laser scanning microscopy, following exposure of Oregon Green-pre-loaded cotyledon sections to (C) a control bath solution or to (D) a bath solution containing 600 μM BAPTA (blue arrow) and replaced with a 3mM CaCl_2_ solution (black double arrow). Schematic diagrams of epidermal cells identifying positions (rectangles) at which relative [Ca^2+^]_cyt_ was monitored (colour coded with traces).

The reproducible presence of fl-DHP fluorescence circumscribing each epidermal cell nucleus (Fig. 3B) suggests that Ca^2+^-permeable channels were located on endomembranes as well as the plasma membrane. In contrast to the even distribution around the cell perimeter of the plasma membrane marker, RH-414, fl-DHP fluorescence was 2.5±0.1 (*n*=60) times more intense along the outer periclinal region of each epidermal cell compared with the remaining cell perimeter and on the inner cytoplasmic edge of each epidermal cell nucleus (Figs 3B; Supplementary S2D, H; Supplementary Table S2 at *JXB* online). These data suggest that the outer periclinal portion of the plasma membrane is enriched in nifedipine-sensitive Ca^2+^-permeable channels and that these channels are essentially absent from the plasma membrane lining anticlinal and inner periclinal walls of each epidermal cell.

Both plasma membrane, and to a lesser extent endomembrane, Ca^2+^-permeable channels were induced upon cotyledon culture (Fig. 3B versus A). That plasma membrane Ca^2+^-permeable channels, asymmetrically localized to the outer periclinal region of each adaxial epidermal cell (Fig. 3B; Supplementary Fig. S2D at *JXB* online; above text), generate the polarized cytosolic Ca^2+^ signal (Fig. 1C, D) is supported by [Ca^2+^]_cyt_ being similarly depressed by BAPTA (Fig. 2G) and the general Ca^2+^ channel blocker, gadolinium (Table 1).

Real-time monitoring demonstrated that [Ca^2+^]_cyt_ in the outer periclinal cytosol was temporally invariant, with no evidence of oscillating back to basal [Ca^2+^]_cyt_ levels (Fig. 3C). The slow decline in Oregon Green fluorescence intensity (0.02% s^–1^), emitted from the outer periclinal cytosol (Fig. 3C), equates with photobleaching rates of Oregon Green recorded by Furch *et al.* (2009).

**Fig. 4.**
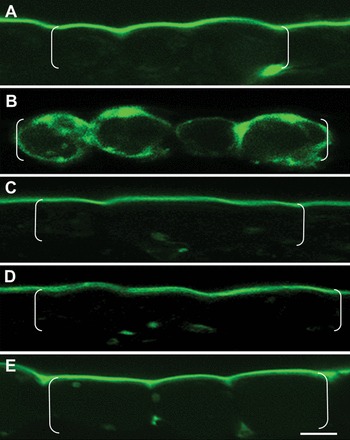
Effect of blocking Ca^2+^-ATPase and Ca^2+^/proton antiporter activity on the intracellular distribution of the Ca^2+^ signal. (A–E) Confocal laser scanning images of Oregon Green fluorescence in transverse sections of cotyledons cultured for 15h in the absence (A) or presence of 500nM Eosin Yellow (B), 100 μM cyclopiazonic acid (C), 1mM ruthenium red (D), and 5 μM bafilomycin A1 (E). Epidermal cells are bracketed. Scale bar=20 μm.

The polarity of the persistent cytosolic Ca^2+^ signal must depend upon minimizing lateral spread of Ca^2+^ throughout the entire cytosol of each epidermal cell. This could be achieved by Ca^2+^ fluxes into, and from, the outer periclinal cytosolic pool being rapid and equally matched; a claim supported by the Ca^2+^ signal intensity reaching new steady-state levels within 72±11 s upon BAPTA chelation of extracellular Ca^2+^ and within 560±71 s upon re-establishing a supply of extracellular Ca^2+^ (Fig. 3D). Consistent with Ca^2+^ signal polarity being dependent upon a rapid Ca^2+^ withdrawal from the cytosol, inhibition of plasma membrane Ca^2+^-ATPase activity with Eosin Yellow caused the Ca^2+^ signal to be dissipated around the entire cytosol of each epidermal cell (Fig. 4B versus A). This led to an estimated 1.9±0.1-fold increase in overall Ca^2+^ content per cell cytosol. In contrast, the polarity of the cytosolic Ca^2+^ signal remained unaltered when endomembrane Ca^2+^-ATPases were inhibited with cyclopiazonic acid. A similar outcome was obtained when Ca^2+^/proton antiport into mitochondria was blocked by ruthenium red or into vacuoles by dissipating the tonoplast proton motive force by inhibiting the vacuolar H^+^-ATPase with bafilomycin A1 (Fig. 4C–E). These data indicate that maintenance of a persistent (Fig. 3C) and polarized cytosolic Ca^2+^ signal (Fig. 4A) can be attributed to the co-operative activities of Ca^2+^-permeable channels and Ca^2+^-ATPases localized to the outer periclinal portion of the plasma membrane of each epidermal cell.

### The polarized Ca^2+^ signal is organized in discrete plumes proximal to the plasma membrane

**Fig. 5.**
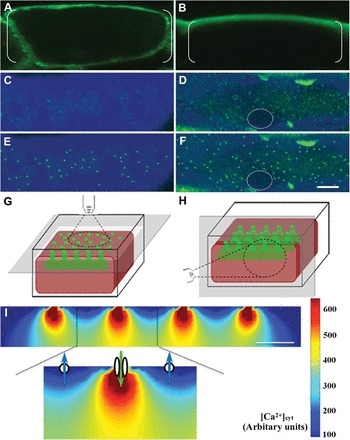
Spatial organization of plasma membrane Ca^2+^-permeable channels, Ca^2+^-ATPases, and the Ca^2+^ signal. (A, B) Transverse sections of epidermal cells illustrating the intracellular distribution of fl-DHP and Oregon Green fluorescence. (C, E) Overlay images of a paradermal section of an epidermal cell co-stained with fl-DHP and Calcofluor White focused on the cell wall–cytoplasm interface. (D, F) Overlay images of a paradermal section of an Oregon Green-loaded epidermal cell counterstained with Calcofluor White focused on the cell wall–cytoplasm interface. The nucleus is outlined by a broken white ring. Discrete patches of fl-DHP (E) and Oregon Green (F) fluorescence, proximal to the cell wall–cytoplasmic interface, highlighted by a computerized algorithm (Supplementary Fig. S3 at *JXB* online). (G, H) Schematic diagrams of adaxial epidermal cells illustrating optical planes at which Orgeon Green fluorescence was visualized in paradermal (G) and transverse (H) sections. (I) Diagrammatic transverse section of an epidermal cell in which Ca^2+^ dynamics have been mathematically modelled to reach equilibrium. Ca^2+^ plumes are generated by the co-operative activities of plasma membrane Ca^2+^-permeable channel clusters influxing Ca^2+^ (inset green arrow between two ovals) and Ca^2+^-ATPases effluxing Ca^2+^ (blue arrows through circles). Inward of the plasma membrane, the Ca^2+^ plumes coalesce into a uniform band. Scale bar = 5 µm for A to F and 1 µm for I.

Imaged in transverse section, fl-DHP (Ca^2+^-permeable channels) and Oregon Green (cytosolic Ca^2+^ signal) fluorescence appeared to be of uniform intensity across the outer periclinal interface of each epidermal cell (Fig. 5A, B, respectively). This spatial organization is not reconcilable with a signal providing positional information to guide deposition of discrete wall ingrowth papillae (Fig. 2E). To investigate further the spatial organization of the polarized Ca^2+^ signal, paradermal cotyledon sections were stained with fl-DHP to determine the lateral organization of Ca^2+^-permeable channels within the plasma membrane lining the outer periclinal portion of each epidermal cell. Imaging epidermal cells in *z*-stacks located their cell wall–cytoplasm interface as a zone of reduced Calcofluor White fluorescence (Fig. 5C) within the mid-region of each dome-shaped outer periclinal cell wall (Fig. 1A, I). fl-DHP fluorescence at these cell wall–cytoplasm interfaces appeared as scattered spots of fluorescence within a matrix of background noise (Fig. 5C). To remove potential image artefacts, the raw images (Fig. 5C) were further analysed using an unbiased computerized algorithm that ensured recognition of near circular fluorescence spots within the background noise (for details, see Supplementary Fig. S4 at *JXB* online). This analysis detected clumps of bright fl-DHP fluorescence at the outer periclinal cell wall–cytoplasm interface (Fig. 5E) consistent with Ca^2+^-permeable channels being organized as discrete clusters within the plasma membrane.

An identical approach to that described above searched for cytosolic Ca^2+^ signals in paradermal sections cut from Oregon Green-pre-loaded cotyledons (Fig. 5D, F). Following analysis of the captured raw images (Fig. 5D), distinct patches of Oregon Green fluorescence were detected proximal to outer periclinal cell wall–cytoplasm interfaces of epidermal cells (Fig. 5F). Based on the above observations, it is hypothesized that the bright patches of Oregon Green fluorescence, viewed in paradermal sections (Fig. 5F), arose from narrow plumes of elevated [Ca^2+^]_cyt_ (Fig. 5G), released by clusters of plasma membrane Ca^2+^-permeable channels (Fig. 5E). In contrast to the fl-DHP fluorescent patches (Fig. 5E), the bright Oregon Green fluorescent patches overlaid a faint, but continuous, spread of fluorescence, except where nuclei are located (Fig. 5D, F). The latter fluorescence was interpreted as arising from the cytosolic Ca^2+^ plumes coalescing at ~500nm inward from the cell wall-cytoplasm interface (Fig. 5G, H). Furthermore, the inward-directed gradient of [Ca^2+^]_cyt_ is consistent with extracellular Ca2+, and not intracellular Ca2+, stores being the source from which the Ca^2+^ signal was derived.

The spatial configuration described above contributed to an optical uniformity of Oregon Green fluorescence when viewed in the confocal *x*/*y*-axis of transverse sections (Fig. 5G, H). This effect is further compounded by the *z*-axis confocal focal plane, with an ideal resolving power of 1000nm. The *z* plane will capture several rows of fl-DHP fluorescent patches or Oregon Green fluorescent plumes (Fig. 5H) rendered non-resolvable at their separation distances of 1000nm (Table 2).

**Table 2.** Diameters of, and distance between, wall ingrowth (WI) papillae, Ca^2+^-permeable channel clusters, and Ca^2+^ plumesData were obtained from scanning electron microscope (e.g. Fig. 2E) and confocal laser scanning microscope (Fig. 5C, D) images.

Feature measured	Diameter (nm)	Distance (nm)
WI papillae	383±6	1579±46
Ca^2+^ channel clusters	317±7	1481±47
Ca^2+^ plumes	326±4	1484±50

Data represent the mean ±SEM determined from observations of 40 cells per cotyledon across four replicates.

The apparent stationary appearance of Oregon Green fluorescent patches (Fig. 5D) in cells undergoing cytoplasmic streaming might be reconciled as follows. Cytoplasmic streaming, flowing at right angles across the plumes, would move dye molecules laterally from regions of high to basal [Ca^2+^]_cyt_ (Fig. 5I). Using a maximal velocity for cytoplasmic streaming of 4.3nm ms^–1^ (Tominaga *et al.*, 2013) and a rate constant of 930nM ms^–1^ for Ca^2+^/Oregon Green association/dissociation (Bortolozzi *et al.*, 2008) predicts that the fluorescence intensity of dye molecules, displaced by cytoplasmic streaming from regions of 600nM to 100nM [Ca^2+^]_cyt_, would decline to basal levels within 2.3nm of entering a 100nM [Ca^2+^]_cyt_ region. This is a non-detectable displacement across a fluorescent patch of 326nm in diameter (Table 2).

Consistent with clusters of plasma membrane Ca^2+^-permeable channels generating cytosolic Ca^2+^ plumes are their comparable diameters and spacing distances (Table 2). Spatial inter-relationships between the Ca^2+^-permeable channels (Fig. 5E) and Ca^2+^-ATPases (Fig. 4B versus A) to form co-operatively a polarized Ca^2+^ signal (Fig. 5B) organized into discrete plumes proximal to the plasma membrane (Fig. 5G, H) were evaluated by a two-dimensional mathematical model (see the Materials and methods). Based on data presented in Table 2, clusters of Ca^2+^-permeable channels were placed at 1.5 μm centres on a hypothetical plasma membrane with the intervening membrane region populated by evenly spaced Ca^2+^-ATPase clusters (Fig. 5I). Running this model until [Ca^2+^]_cyt_ reached steady levels reproduced the predicted *in vivo* configuration of a polarized Ca^2+^ signal comprised of discrete cytosolic Ca^2+^ plumes proximal to the plasma membrane, whilst, inward of this point, [Ca^2+^]_cyt_ merged into a uniform distribution (Fig. 5I).

### A polarized cytosolic Ca^2+^ signal, organized into discrete plumes, selectively regulates deposition of wall ingrowth papillae but not the uniform wall

**Fig. 6.**
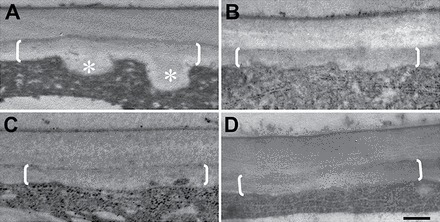
Dependence of ingrowth wall formation on a Ca^2+^ signal. (A–D) Representative transmission electron microscope images of transverse sections of the outer periclinal region of cotyledon epidermal cells cultured on MS medium alone (A), and MS medium containing 100 μM nifedipine (B), 500nM Eosin Yellow (C), or 100 μM A23187 (D). The uniform wall is bracketed (A–D) and wall ingrowth papillae are labelled with asterisks (A). Scale bar=250nm.

Similar diameters and separation distances (Table 2) between Ca^2+^ plumes and wall ingrowth papillae suggest the Ca^2+^ plumes provide positional information to direct the deposition of wall ingrowth papillae. This hypothesis was tested by employing two approaches that were found to obliterate the Ca^2+^ plumes without dampening cytosolic Ca^2+^ levels. These approaches were: (i) blocking Ca^2+^ efflux from the epidermal cells (see Fig. 5I) by inhibiting the plasma membrane Ca^2+^-ATPases with Eosin Yellow (Fig. 4B versus A); and (ii) flooding the epidermal cells with Ca^2+^ by exposing them to the Ca^2+^ ionophore, A23187 (Supplementary Fig. S5B versus A at *JXB* online). Under these conditions, deposition of wall ingrowth papillae was abolished whilst uniform wall formation of the ingrowth wall was unaltered (Table 3; Fig. 6B, C versus A). Similarly, when the Ca^2+^ signal was attenuated by exposing cotyledons to nifedipine (Table 1), wall ingrowth deposition was blocked without compromising construction of the uniform wall (Table 3). Together, these data demonstrate that the polarized plumes of the cytosolic Ca^2+^ signal (Fig. 5I) selectively direct localized construction of wall ingrowth papillae (Table 3; Fig. 6A) whilst exerting no influence over uniform wall formation (Table 3; Fig. 6). Also consistent with this conclusion is the finding that, in the absence of blocking Ca^2+^ signal generation with inhibitors of endomembrane-localized Ca^2+^-ATPases (Fig. 4C versus A) or Ca^2+^/proton antiporters (Fig. 4D versus A), there was no effect on epidermal cells forming wall ingrowth papillae (Supplementary Table S3).

**Table 3.** Impact of obliterating the Ca^2+^ signal on uniform wall and formation of wall ingrowth papillae (WI)

Cotyledon treatment	Uniform wall thickness (nm)	% of cells with WIs
Control	234±14	86.5±1.8
Eosin Yellow (500nM)	232±7	17.9±1.9
A23187 (100 μM)	223±6	10.7±0.9
Nifedipine (100 μM)	243±6	34.5±2.0

Mean ±SEM of 10 cells per cotyledon across six replicates.

## Discussion

The present work has identified a polarized cytosolic Ca^2+^ signal that is temporally invariant but spatially complex in fully expanded cotyledon epidermal cells *trans*-differentiating to a transfer cell morphology. The cell-specific Ca^2+^ signal selectively functions to direct deposition of cell wall material to discrete loci, located on the outer periclinal walls of the *trans*-differentiating epidermal cells, for the construction of wall ingrowth papillae.

To date, cytosolic Ca^2+^ signals, which are known to regulate plant development, establish symbiotic partnerships and orchestrate responses to biotic or abiotic stresses, invariably are structured as single or oscillating spikes with periodicities ranging from seconds to minutes (Kudla *et al.*, 2010; Reddy *et al.*, 2011). Similar temporal periodicities have been observed for cytosolic Ca^2+^ signals formed in algae (Wheeler and Brownlee, 2008), fungal hyphae (Brand and Gow, 2009), and animal cells (Leybaert and Sanderson, 2012). In contrast, once established in cotyledon epidermal cells (Supplementary Fig. S1F versus B at *JXB* online), the cytosolic Ca^2+^ signal exhibited temporal invariance for up to 1h (Fig. 3C). Thus, information encrypted in the epidermal cell cytosolic Ca^2+^ signal probably relies on its structural organization that exhibited two key characteristics. First it was polarized to the outer periclinal region of each epidermal cell (Figs 1D, 2B, 4A, 5B). Secondly, the cytosolic Ca^2+^ signal was organized into discrete plumes proximal to the plasma membrane–cytoplasm interface (Fig. 5F, I).

Generation of a spatial cytosolic Ca^2+^ signal results from activities of Ca^2+^-permeable channels supporting a Ca^2+^ flux into a cell’s cytosol from extra- and/or intracellular compartments co-ordinated with those of Ca^2+^-ATPases and Ca^2+^/proton antiporters withdrawing cytosolic Ca^2+^ back into these compartments to provide temporal shape to the signal (Wheeler and Brownlee, 2008; Kudla *et al.*, 2010; Hepler *et al.*, 2012). During culture, Ca^2+^-permeable channels in cotyledon epidermal cells were enriched in portions of plasma membrane lining their outer periclinal walls and endomembranes (Fig. 3A versus B; Supplementary Fig. S3B at *JXB* online). As found for tip growth systems (Wheeler and Brownlee, 2008; Brand and Gow, 2009; Kudla *et al.*, 2010; Hepler *et al.*, 2012), a flow of extracellular Ca^2+^ (Fig. 2) into the cytosol of epidermal cells through plasma membrane Ca^2+^-permeable channels (Fig. 3B, Supplementary Fig. S3B versus D) plays a major role in establishing the polarized Ca^2+^ signal (Table 1). Since the widths of the outer periclinal cytosol correspond to those of the cytosolic Ca^2+^ signals (i.e. 940nm in width), it is likely that the inner boundary of the cytosolic Ca^2+^ signal is constrained by the tonoplast of each epidermal cell. Restriction of the cytosolic Ca^2+^ signal to the outer periclinal region of each epidermal cell cytosol (Figs 1D, 2B, 4A, 5B) is accounted for by rapid withdrawal rates of Ca^2+^ from this compartment (Fig. 3D), by plasma membrane Ca^2+^-ATPases (Fig. 4B versus A) located at the corners between the outer periclinal and anticlinal cell walls (Fig. 5I).

A unique feature of the polarized cytosolic Ca^2+^ signal formed in each epidermal cell (Figs 1D, 2B, 4A, 5B) was that its substructure is organized into spatially discrete plumes proximal to plasma membrane lining their outer periclinal walls, as demonstrated experimentally (Fig. 5F) and confirmed by modelling (Fig. 5I). The cytosolic Ca^2+^ plumes arose from fluxes of extracellular Ca^2+^ entering the cytosol of each epidermal cell through clusters of plasma membrane Ca^2+^-permeable channels (Fig. 5E) localized to their outer adaxial region (Figs 3B, 5A; Supplementary Table S2 at *JXB* online). Mathematical modelling confirmed this scenario as well as highlighting the co-operative role of plasma membrane Ca^2+^-ATPases in depleting the elevated [Ca^2+^]_cyt_ between the Ca^2+^-permeable channel clusters to create the discrete plumes of cytosolic Ca^2+^ (Fig. 5I). This plasma membrane organization is analogous to that found for animal cells where Ca^2+^-permeable channels are clustered into plasma membrane microdomains to orchestrate specific spatiotemporal Ca^2+^ signals (Pani and Sing, 2009). Although the idea of clustering of Ca^2+^ channels has been proposed a mechanistic basis for localized-mediated Ca^2+^ signalling (Trewavas and Mahló, 1997), it is only recently that evidence for this phenomenon has emerged. For example, Ca^2+^ hot spots have been proposed to arise within sieve element lumens from observed localized groupings of plasma membrane Ca^2+^-permeable channels aggregated around orifices of branched pore plasmodesmal units interconnecting sieve elements with their adjoining companion cells (Furch *et al.*, 2009). However, the present work represents the first report of Ca^2+^-permeable channels being compartmented as clusters surrounded by, aggregates of Ca^2+^-ATPase in the plasma membrane of a plant cell to create persistent plumes of cytosolic Ca^2+^. Significantly, the estimated diameters of these Ca^2+^-permeable channel clusters (Table 2) fall into the size range reported for microdomains found in plant cells (Malinsky *et al.*, 2013).

**Fig. 7.**
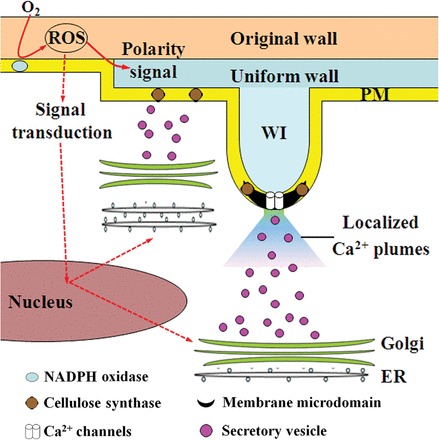
Schematic model of the signalling cascade regulating ingrowth wall formation. Ethylene-induced extracellular reactive oxygen species (ROS) production activates the cell wall biosynthesis machinery and provides a positional cue to determine the polarity of uniform wall deposition. Localized Ca^2+^ plumes, formed by the co-operative activity of plasma membrane Ca^2+^-permeable channel clusters and Ca^2+^-ATPases, create loci that determine sites at which wall ingrowth (WI) papillae are constructed. PM, plasma membrane; ER, endoplasmic reticulum.

This work provides insight into how the cytosolic Ca^2+^ signal, described above, regulates deposition of ingrowth walls in epidermal cells of cultured cotyledons *trans*-differentiating to a transfer cell morphology (Fig. 7). Formation of their polarized ingrowth walls is a two-step process involving polarized deposition of a distinctive uniform wall on which wall ingrowth papillae subsequently are constructed at discrete loci (McCurdy *et al.*, 2008). An ethylene-induced polarized extracellular reactive oxygen species (ROS) signal initiates wall biosynthesis and exerts directional influence over cellular positioning of uniform wall deposition exclusively to the outer periclinal wall of each cotyledon epidermal cell (Andriunas *et al.*, 2012; Xia *et al.*, 2012) (Fig. 7). However, contrary to the ubiquitous central influence of a polarized cytosolic Ca^2+^ signal regulating tip growth (Wheeler and Brownlee, 2008; Brand and Gow, 2009; Kudla *et al.*, 2010), the current findings suggest that cytosolic Ca^2+^ plays, at best, a secondary role in uniform wall formation and positioning (Table 3; Fig. 6) whilst ROS signalling exerts a dominant influence (Andriunas *et al.*, 2012; Xia *et al.*, 2012). In contrast, deposition of wall ingrowth papillae at discrete loci on the uniform wall layer was found to be dependent upon, and directed by, discrete plumes of cytosolic Ca^2+^. Evidence for this assertion includes an absence of wall ingrowth papillae when cytosolic Ca^2+^ plumes are removed by slowing Ca^2+^ influx by depleting extracellular Ca^2+^ with the Ca^2+^ chelator, BAPTA (Fig. 2F versus E, G), or by blocking Ca^2+^ channel activity (Table 1), or are obliterated by flooding the epidermal cell cytosol with excess Ca^2+^ following exposure to Eosin Yellow or A23187 (Table 3; Figs. 4B; Supplementary Fig. S4B at *JXB* online). Further evidence consistent with this assertion includes the finding that densities (Figs 2E, 5E, F), diameters of, and distance between Ca^2+^-permeable channels, cytosolic Ca^2+^ plumes, and wall ingrowth papillae closely correspond (Table 2). Thus, cytosolic Ca^2+^ plumes (Fig. 5F, I) impart spatial information to form loci that direct deposition of wall ingrowth papillae possibly through re-organizing the actin cytoskeleton (Fig. 7).

What is not certain is whether the Ca^2+^ plumes only provide positional information to direct delivery of cell wall matrix polysaccharides and plasma membrane-localized cell wall biosynthetic enzymes (cellulose synthases, callose synthases, and glucanases) to loci at which wall ingrowths are constructed. An additional role for the Ca^2+^ plumes could be to regulate the catalytic activity of plasma membrane-localized cell wall biosynthetic enzymes located at these loci. For instance, within sieve elements, putative Ca^2+^ hot spots have been shown to regulate the localized synthesis of callose deposits (Furch *et al.*, 2009). In this context, the Ca^2+^ plumes could elicit localized post-translational activation of callose synthases positioned along the plasma membrane lining the outer periclinal wall of each epidermal cell. The resulting callose deposits provide a plastic matrix in which cellulose microfibrils, extruded from co-localized clusters of cellulose synthases, reach a rigid crystalline state before encountering the counter force of the non-deformable rigid wall (Diotallevi and Mulder, 2007). This scenario is consistent with the substructure of wall ingrowth papillae, comprising an inner core of cellulose microfibrils, orientated in whorls perpendicular to the uniform wall, and enshrouded by a substantive callose sheath (Talbot *et al.*, 2007; Vaughn *et al.*, 2007). The absence of any detectable change in uniform wall thickness when deposition of wall ingrowth papillae was blocked upon dissipating the Ca^2+^ plumes but not the elevated [Ca^2+^]_cyt_ (Table 3), that would sustain an active callose deposition (Furch *et al.*, 2009), can be accounted for by the fact that the total volume of wall ingrowth papillae is only 1% of the uniform wall volume (estimated from data presented in Tables 2 and 3). Thus, if cell wall biosynthesis continued in the absence of the Ca^2+^ plumes, the contribution to uniform wall thickness would not be detectable.

In conclusion, a novel cytosolic Ca^2+^ signal comprised of temporally stable but spatially localized plumes, generated by the co-operative activities of plasma membrane clusters of Ca^2+^-permeable channels surrounded by aggregates of Ca^2+^-ATPases, direct the localized deposition of wall ingrowth papillae in epidermal cells *trans-*differentiating to a transfer cell morphology.

## Supplementary data

Supplementary data are available at *JXB* online

Figure S1. Effects of cotyledon culture time, Oregon Green loading temperature, and cell viability on the formation of detectable Oregon Green fluorescence in adaxial epidermal cells of *V. faba* cotyledons.

Figure S2. Subcellular localization of fl-DHP fluorescence in adaxial epidermal cells of *V. faba* cotyledons cultured on MS medium.

Figure S3. Competitive effects of non-labelled nifedipine on fl-DHP fluorescence.

Figure S4. A three-dimensional reconstructed fluorescence intensity profile, generated by a computerized algorithm, of a fluorescent patch captured from a CLSM image of a paradermal cotyledon section labelled with fl-DHP or OGB-1.

Figure S5. Intracellular distribution of the Ca^2+^ signal in adaxial epidermal cells of *V. faba* cotyledons.

Table S1. Intracellular distribution of Oregon Green 488 BAPTA-1 and hydroxypyrene-1,3,6-trisulphonic acid, trisodium (HPTS) in, together with cytoplasmic volumes of outer and inner periclinal regions of, epidermal cells of cultured cotyledons.

Table S2. Competitive effect of nifedipine on intracellular distribution of fl-DHP, RH-414 fluorescence in epidermal cells of cultured cotyledons.

Table S3. Effect of blockers of endomembrane Ca^2+^-ATPases (thapsigargin, cyclopiazonic acid) and Ca^2+^/proton antiporters (bafilomycin A1) on wall ingrowth papillae formation.

Supplementary Data

## Abbreviations:

2-APB: 2-aminoethoxydiphenyl borate
AM: acetoxymethyl
BAPTA: 1, 2-bis(*o*-aminophenoxy)ethane-*N*,*N*,*N*’,*N*’-tetraacetic acid
[Ca^2+^]_cyt_: cytosolic Ca^2+^ concentration
CCCP: carbonyl cyanide *m*-chlorophenyl hydrazine
CLSM: confocal laser scanning microscope
ER: endoplasmic reticulum
fl-DHP: DM-BODIPY(–)-dihydropyridine
HPTS: 8-acetoxypyrene-l,3,6-trisulphonic acid
H_2_O_2_: hydrogen peroxide
MS: Murishige and Skoog
PBS: phosphate-buffered saline
PM: plasma membrane
RH-414: *N*-(3-triethylammoniumpropyl)-4-(4-(4-diethylamino)phenyl)butadienyl)pyridinium dibromide
SEM: standard error of the mean.
