# Supplementary material for: Polarized and persistent Ca2+ plumes define loci for formation of wall ingrowth papillae in transfer cells
Source: J Exp Bot. 2014 Dec 10;66(5):1179–90. doi: 10.1093/jxb/eru460 (PMC4339585; doi:10.1093/jxb/eru460)
Supplement: Supplementary Data [file supp_66_5_1179__index.html]

Polarized and persistent Ca2+ plumes define loci for formation of wall ingrowth papillae in transfer cells — Polarized and persistent Ca2+ plumes define loci for formation of wall ingrowth papillae in transfer cells — Supplementary Data 

# Polarized and persistent Ca2+ plumes define loci for formation of wall ingrowth papillae in transfer cells

## Supplementary Data

Data files

**Files in this Data Supplement:**

- Supplementary Data - Supplementary Data
