# Supplementary material for: Polarized and persistent Ca2+ plumes define loci for formation of wall ingrowth papillae in transfer cells
Source: J Exp Bot. 2014 Dec 10;66(5):1179–90. doi: 10.1093/jxb/eru460 (PMC4339585; doi:10.1093/jxb/eru460)
Supplement: Supplementary Data [file supp_eru460_jexbot127944_file001.pdf]

**Supplementary data - Figures**

# Polarized and persistent $Ca^{2+}$ plumes define loci for wall ingrowth papillae formation in transfer cells

Hui-Ming Zhang, Mohammad S Imtiaz, Derek R Laver, David W McCurdy, Christina E Offler, Dirk F van Helden, and John W Patrick

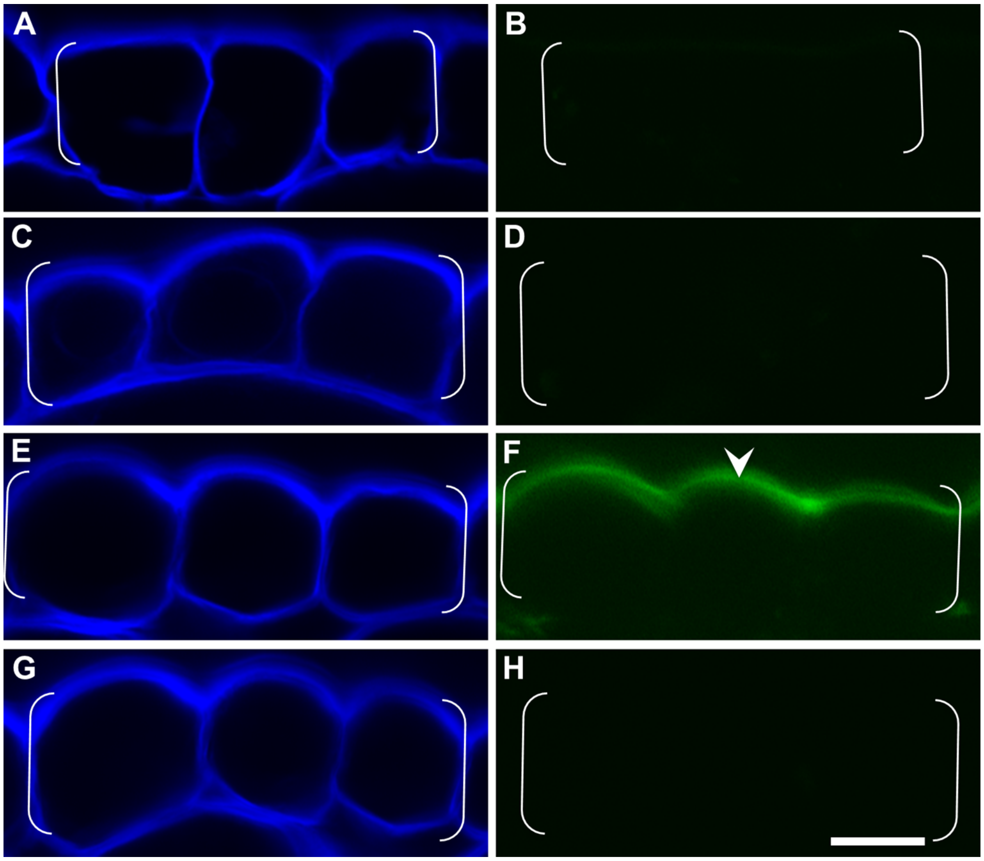


**Fig. S1**. Effects of cotyledon culture time, Oregon Green loading temperature and cell viability on the formation of detectable Oregon Green fluorescence (arrowhead) in adaxial epidermal cells (identified within brackets) of *V. faba* cotyledons. Confocal laser scanning images of transverse sections of epidermal cells stained with Calcofluor White (A, C, E, G) and the corresponding cells pre-loaded with Oregon Green (B, D, F, H). Cotyledons were freshly-harvested (A, B) or cultured for 15 h (C-H) on MS medium alone (C-F) or on MS medium containing 600 $\mu$M sodium azide (G, H). Oregon Green pre-loading was performed at 26 ºC (C, D) or 4 ºC (A, B; E, F, G, H). Note the $Ca^{2+}$ signal was only present in viable cells pre-loaded with Oregon Green at 4 ºC (F). Oregon Green loading at 4 ºC avoided Oregon Green AM ester de-esterification by cell wall esterases which occurred at 26 ºC. De-esterification by cell wall esterases prevented transport of the charged de-esterified fluorochrome into cells across their plasma membrane (D). (Scale bar 20 $\mu$m).

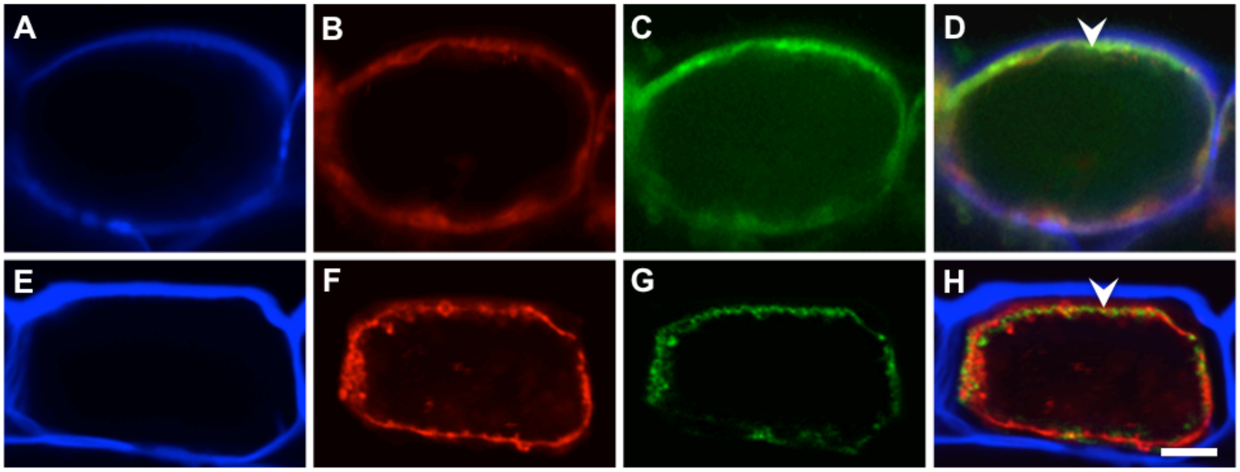


**Fig. S2**. Sub-cellular localization of fl-DHP fluorescence in adaxial epidermal cells of *V. faba* cotyledons cultured on MS medium. Confocal laser scanning images of transverse sections of turgid (A–D) and plasmolyzed (E–H) adaxial epidermal cells. Visualized are their cell walls (blue fluorescence of Calcofluor White stain; A, E), plasma membranes (red fluorescence of plasma membrane marker, RH-414; B, F) and $Ca^{2+}$ channels (green fluorescence of fl-DHP; C, G) together with their image overlays (D, H). In turgid cells (D), the fl-DHP fluorescence coincides with the plasma membrane fluorescence by RH-414 (arrowhead in D) except around the inner portion of each nucleus (arrow in D) where only the fl-DHP fluoresence was apparent (C vs B). In plasmolyzed cells (H), the overlap between fl-DHP and RH-414 fluorescence was retained in protoplasts that had retracted from their cell walls (arrowhead in H). The overlap between turgid (D) and plasmolyzed (H) epidermal cells indicated that both signals were associated with epidermal cell protoplasts. (Scale bar 10 $\mu$m).

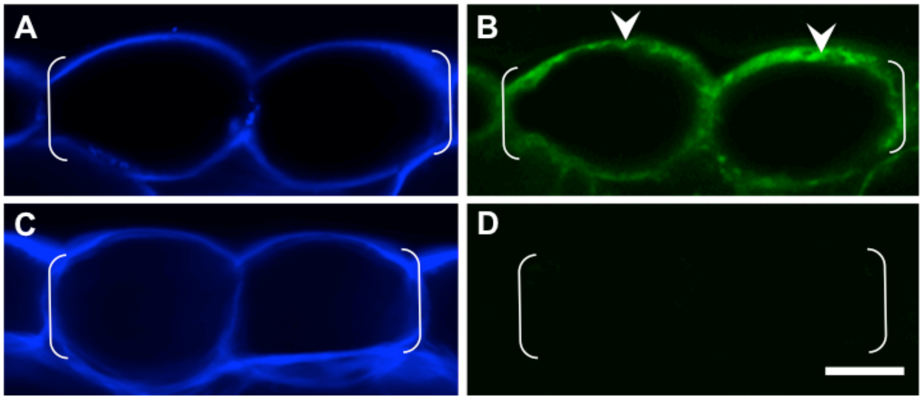


**Fig. S3.** Competitive effects of non-labelled nifedipine on fl-DHP fluorescence. Confocal laser scanning images of transverse sections of adaxial epidermal cells co-stained with Calcofluor White (A, C) and fl-DHP (B, D) with (C, D) or without (A, B) 500 nM nifedipine. (Scale bar 20 $\mu$m).

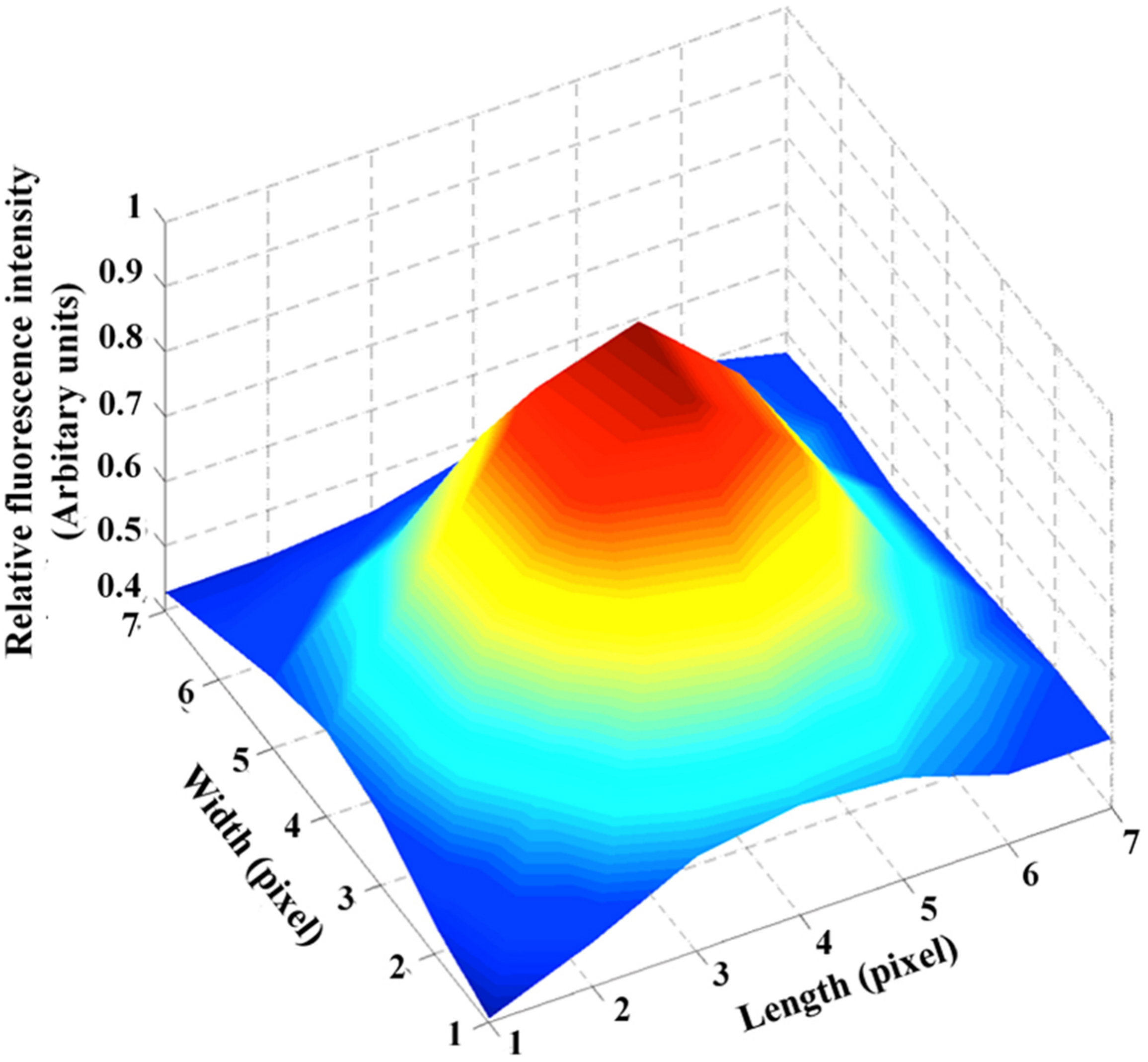


**Fig. S4.** A three-dimensional reconstructed fluorescence intensity profile, generated by a computerized algorithm, of a fluorescent patch captured from a CLSM image of a paradermal cotyledon section labelled with fl-DHP or OGB-1 (see Fig. 5C, D). Genuine fluorescent patches were distinguished from background noise by determining whether their fluorescent intensity profiles complied with a point-spread function (for more details, see Materials and Methods).

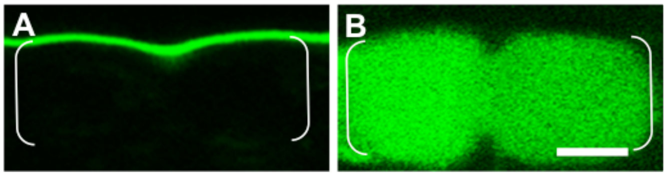


**Fig. S5.** Intracellular distribution of the $Ca^{2+}$ signal in adaxial epidermal cells (bracketed) of *V. faba* cotyledons. Cotyledons were cultured in the absence (A) or presence of 100 $\mu$M A23187 (B). Confocal laser scanning images of transverse sections of cultured cotyledons pre-loaded with Oregon Green. Note the polarized Oregon Green fluorescence (A) was dispersed uniformly throughout cells exposed to A23187 (B). (Scale bar 20 $\mu$m).

**Supplementary data - Tables**

# Polarized and persistent $Ca^{2+}$ plumes define loci for wall ingrowth papillae formation in transfer cells

Hui-Ming Zhang, Mohammad S Imtiaz, Derek R Laver, David W McCurdy, Christina E Offler, Dirk F van Helden, and John W Patrick

**Table S1.** Intracellular distribution of Oregon Green 488 BAPTA-1 and hydroxypyrene-1,3,6-trisulphonic acid, trisodium (HPTS) in, together with cytoplasmic volumes of outer and inner periclinal regions of, epidermal cells of cultured cotyledons. Intracellular distribution of Oregon Green was evaluated by uniformly elevating $[Ca^{2+}]_{cyt}$ throughout the epidermal cell cytosol by permeabilizing cells with the $Ca^{2+}$ ionophore, A23187, or by blocking $Ca^{2+}$ efflux from the cells with the plasma membrane $Ca^{2+}$-ATPase inhibitor, Eosin Yellow.

| **Parameter measured** | **Treatment** | **Sub-cellular location** | |
|---|---|---|---|
| | | **Outer periclinal** | **Inner periclinal** |
| Oregon Green fluorescence intensity (pixel intensity) | Control | 81.2 ± 3.2 | 6.7 ± 0.9 |
| | 10 μM A23187 | 92.7 ± 2.9 | 89.4 ± 2.9 |
| | 0.5 μM Eosin Yellow | 92.7 ± 2.7 | 89.8 ± 3.0 |
| HPTS fluorescence intensity (pixel intensity) | Control | 43.5 ± 2.0 | 42.1 ± 1.6 |
| Cytoplasmic volume ($\mu m^3$) | Fresh cotyledons | 432 ± 39 | 427 ± 38 |
| | 15-h cultured cotyledons | 576 ± 37 | 578 ± 34 |

Mean ± s.e.m. Fluorescence intensity measures were of 100 cells from four cotyledons, 20 to 30 cells per cotyledon. Cytoplasmic volume estimates of outer and inner periclinal regions were derived from the product of their areas (400 cells; 100 cells per cotyledon across four replicate cotyledons) and thicknesses (60 cells; 10 cells per cotyledon x six replicate cotyledons).

**Table S2.** Competitive effect of nifedipine on intracellular distribution of fl-DHP, RH-414 fluorescence along with competitive effect of nifedipine on fl-DHP fluorescence in epidermal cells of cultured cotyledons. Fluorescence measured as pixel intensities.

| **Parameter measured** | **Treatment** | **Sub-cellular location** | | |
|---|---|---|---|---|
| | | **Outer periclinal** | **Inner periclinal** | **Nucleus** |
| fl-DHP fluorescence intensity (pixel intensity) | 0.6 μM fl-DHP | 57.6 ± 2.2 | 21.2 ± 0.7 | 22.3 ± 0.8 |
| | 0.1 mM nifedipine + 0.6 μM fl-DHP | 0.6 ± 0.1 | 0.5 ± 0.1 | N/A |
| RH-414 fluorescence intensity (pixel intensity) | Control | 36.8 ± 1.7 | 34.2 ± 1.7 | N/A |

Mean ± s.e.m. Fluorescence intensity measures were of 100 cells from four cotyledons, 20 to 30 cells per cotyledon.

**Table S3.** Effect of blockers of endomembrane $Ca^{2+}$-ATPases (thapsigargin, cyclopiazonic acid) and $Ca^{2+}$/proton antiporters (bafilomycin A1) on wall ingrowth papillae (WI) formation.

| **Cotyledon treatment** | **% cells with WIs** |
|---|---|
| Control | 91.4 ± 1.3 |
| Thapsigargin (5 μM) | 89.1 ± 2.2 |
| Cyclopiazonic acid (100 μM) | 85.3 ± 3.5 |
| Bafilomycin A1 (5 μM) | 88.9 ± 2.3 |

Mean ± s. e. m. of 600 cells from six replicate cotyledons.
